# A Case of Miliary Tuberculosis Complicated by Thyroid Involvement: Managing Rifampicin-Induced Thrombocytopenia With Rifabutin

**DOI:** 10.7759/cureus.57876

**Published:** 2024-04-08

**Authors:** Akimichi Nagashima, Tomoko Kobori, Mototaka Hattori, Shingo Imura, Yasumi Okochi

**Affiliations:** 1 Department of Respiratory Medicine, Japan Community Health Care Organization Tokyo Yamate Medical Center, Tokyo, JPN

**Keywords:** rifampicin, rifampicin induced thrombocytopenia, rifabutin, thyroid tuberculosis, miliary tuberculosis

## Abstract

This case report presents an unusual occurrence of miliary tuberculosis with thyroid tuberculosis in a 75-year-old male patient, who successfully completed the treatment with rifabutin after rifampicin-induced thrombocytopenia. The patient has been suffering from diabetes mellitus and chronic heart failure, and had coronavirus disease of 2019 (COVID-19) just before being diagnosed with miliary tuberculosis. The patient had not been prescribed immunosuppressants and steroids. Chest computed tomography (CT) scans revealed multiple tiny nodules diffusely and equally distributed in bilateral lung fields. Subsequently, polymerase chain reaction (PCR) techniques on the urine samples and culture of sputum demonstrated positivity for *Mycobacterium tuberculosis*. Thus, we conclusively identified miliary tuberculosis and initiated treatment using anti-tuberculosis drugs. During treatment, the patient developed thyroid tuberculosis, resulting in an enlarged thyroid and hoarseness, but these symptoms improved with continued use of the anti-tuberculosis drugs. Moreover, regarding treatment, the rifabutin dosage was completed after changing drugs due to rifampicin-induced thrombocytopenia. Notably, miliary tuberculosis is rarely complicated by thyroid tuberculosis as a paradoxical reaction, and the substitution of rifabutin for rifampicin-induced thrombocytopenia is not fully studied. We present this case alongside relevant prior data for comprehensive clinical insight.

## Introduction

Miliary tuberculosis (TB) is a rare and extremely severe status of TB characterized by the widespread of *Mycobacterium tuberculosis* throughout the body [1]. While miliary TB was previously considered a disease affecting predominantly infants and children, over the past three decades, its recognition has expanded to include adults. The mortality rate related to miliary TB is about 25% to 30% in adults [2]. As delay in diagnosis and the initiation of treatment appears to lead to a higher mortality rate, clinicians should maintain a low threshold for suspecting miliary TB to facilitate early diagnosis and treatment.

Miliary TB can present with a diverse range of clinical manifestations. This case report describes a rare condition: miliary TB complicated by thyroid involvement in an immunocompetent adult. Additionally, the patient underwent treatment with a multidrug regimen of anti-tuberculosis drugs, during which rifampicin-induced thrombocytopenia occurred. Rifabutin was subsequently used to complete the treatment without significant adverse events. Our findings will provide valuable insights into the management of the treatment of miliary TB.

## Case presentation

**Figure 1:**
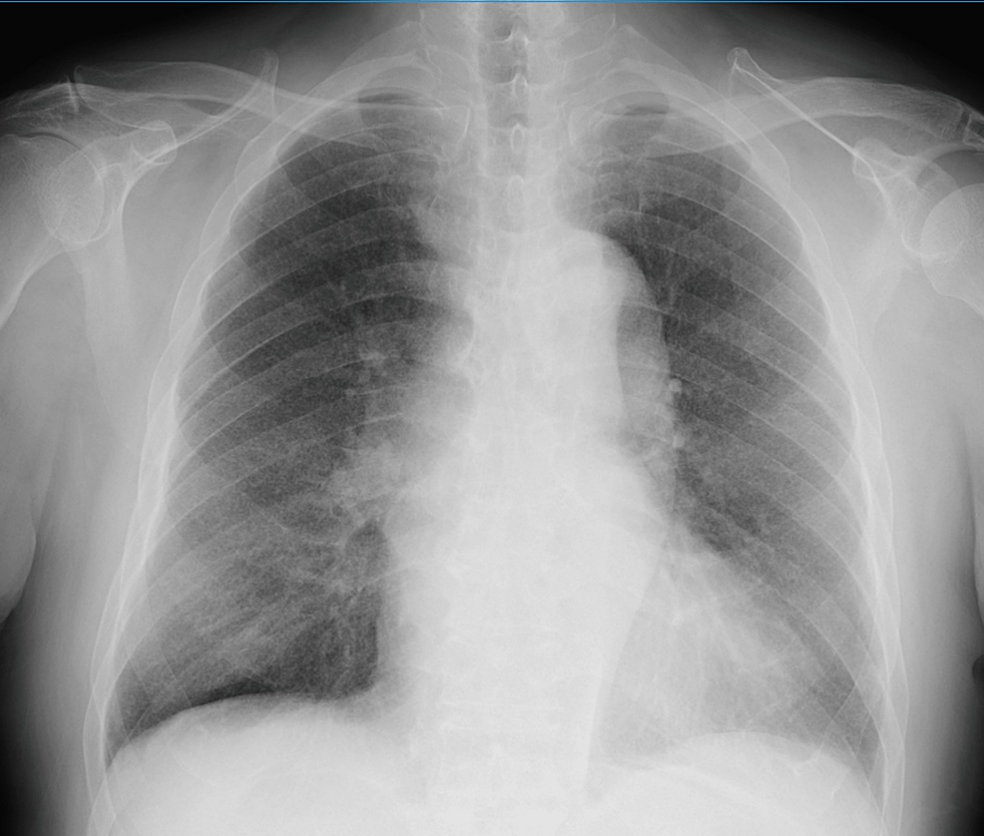
Chest X-ray Chest X-ray showing diffuse bilateral miliary nodules.

We describe the case of a 75-year-old man who presented with several weeks of fever, cough and sputum. At the previous clinic, the patient was diagnosed with the coronavirus disease 2019 (COVID-19). He rested and stayed at his home, but the symptoms continued. Multiple tiny nodular infiltrates in bilateral lung fields were observed in chest X-ray when he came to our hospital (Figure 1). Physical examination of the skin, abdomen, and cardiac exam revealed normal findings. The patient had a history of diabetes mellitus and chronic heart failure, both of which were under treatment, but no other significant medical history. He had no recent travel, no exposure to environmental hazards, and no history of immunosuppressant or steroid use prior to the onset of symptoms.

**Figure 2:**
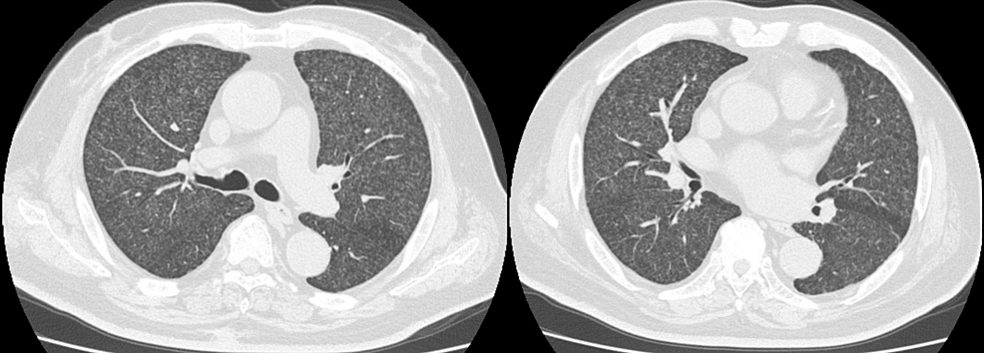
Chest CT Chest CT showing diffuse multiple tiny nodules in bilateral lungs.

The patient was admitted to our hospital and various tests were performed. Initial blood tests indicated a white blood cell (WBC) of 5,200/µL and C-reactive protein (CRP) of 7.1 mg/dL. The interferon-gamma release assays (T-SPOT®.TB) and the human immunodeficiency virus (HIV) antigen and antibody tests returned negative results (Table 1). A chest computed tomography (CT) revealed diffuse, tiny nodules equally distributed in bilateral lung fields (Figure 2). Although sputum smear tests for acid-fast bacteria returned negative, genetic testing of sputum and urine confirmed *M. tuberculosis* through a positive tuberculosis-polymerase chain reaction (PCR). Based on these findings, we diagnosed the patient with miliary TB. Subsequently, combination therapy with rifampicin, isoniazid, ethambutol, and pyrazinamide was initiated. Following the start of treatment, the patient’s fever subsided, and symptoms gradually alleviated. After confirming the absence of any acute adverse events, the patient was discharged. Sputum cultures revealed *M. tuberculosis*, which was susceptible to anti-tuberculosis drugs.

**Table 1:** Laboratory values

Parameter	Value	Reference range
White blood cell count	5.2 × 10^3^/µL	3.5 – 9.0 × 10^3^/µL
Neutrophils	83.2%	37.0 – 72.0%
Eosinophils	0%	0.0 – 5.0%
Hemoglobin	15.1 g/dL	14.0 – 18.0 g/dL
Platelet count	12 × 10^4^/µL	12.0 – 36.0 × 10^4^/µL
Creatinine	1.03 mg/dL	0.65 – 1.07 mg/dL
Blood urea nitrogen	14 mg/dL	8 – 20 mg/dL
Sodium	137 mEq/L	135 – 145 mEq/L
Potassium	4.4 mEq/L	3.4 – 5.0 mEq/L
Albumin	3.3 g/dL	3.9 – 4.9 g/dL
C-reactive protein (CRP)	7.1 mg/dL	0.0 – 0.4 mg/dL
Beta-D glucan	≦4 pg/mL	<11.0 pg/mL
T-SPOT.TB test	Negative	Negative
Human immunodeficiency virus (HIV) antigen and antibody	Negative	Negative

The treatment was continued at the outpatient clinic. However, a month after the initiation of treatment, the platelet count in the blood test began to decrease, reaching 42,000/µL. The drugs were temporarily suspended, and subsequent blood tests were conducted. Once the platelet count had recovered, treatment was resumed with the addition of levofloxacin to ethambutol and pyrazinamide, followed by isoniazid. This time, the platelet count remained stable, and we identified rifampicin as the likely cause of thrombocytopenia. Subsequently, rifabutin was substituted for rifampicin, and treatment was restarted with rifabutin, isoniazid, ethambutol, and pyrazinamide. The platelet count maintained a range between about 50,000 and 100,000/μL and did not decrease. The patient received four drugs for the initial month and continued with rifabutin and isoniazid for an additional seven months. Notably, no significant adverse events were observed during the period.

**Figure 3:**
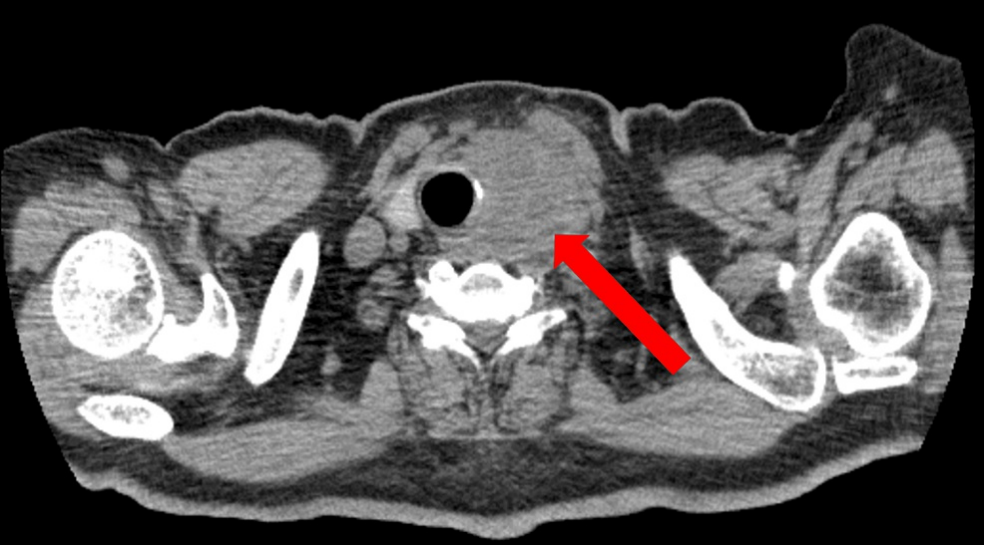
Neck CT Neck CT showing enlargement of the left lobe of the thyroid gland (red arrow).

As the treatment progressed, the patient developed hoarseness and swelling in the left anterior neck approximately two months after initiating the treatment. A CT scan revealed enlargement of the left lobe of the thyroid gland (Figure 3), while ultrasonography confirmed enlargement of the left lobe of the thyroid gland and enlarged lymph nodes.

Additionally, an imbalance of thyroid hormones was observed where only Thyroid-stimulating hormone (TSH) levels were slightly decreased while free triiodothyronine (T3) and thyroxine (T4) levels were within normal range. Fine needle aspiration cytology (FNAC) showed no malignant cells, and the acid-fast bacteria culture returned negative, despite a positive smear for acid-fast bacteria. Considering the course of the disease, thyroid TB was suspected, and the patient’s treatment with anti-tuberculosis drugs was continued. Over the course of the treatment, the swollen thyroid gland and cervical lymph nodes gradually decreased in size, hoarseness alleviated, and the TSH level normalized. Furthermore, diffuse tiny nodules observed on chest CT improved and eventually disappeared. The patient is currently in the observation period post-treatment, and there has been no recurrence to date. 

## Discussion

Miliary TB represents a form of *M. tuberculosis* infection with persistently high mortality rates. The characteristic miliary pattern observed on chest radiographs serves as a classical feature and is often the initial indicator suggestive of miliary TB [2]. In addition to the identification of a diffuse miliary infiltrate on chest radiographs or chest high-resolution CT scans (HRCT), the diagnosis of miliary TB requires histopathological evidence of miliary tubercles in tissue specimens obtained from various organs [2]. A previous study showed the possibility of the effectiveness of using PCR techniques for detecting *M. tuberculosis* in urine samples [3]. Although it was a retrospective study conducted at a single facility, the authors demonstrated positive results by PCR using urine samples in 78.6% of the included patients with suspected and clinical miliary TB [3]. The utilization of PCR techniques and urine sample cultures, alongside sputum analysis, may offer a useful diagnostic method for miliary TB. Moreover, this approach is convenient and safe for patients. We demonstrated the PCR method using urine samples for the detection of *M. tuberculosis* in this case.

The common involvement of miliary TB is organs with high blood flow including the liver, spleen, lungs, and bone marrow. The rate of thyroid involvement in miliary TB varies in previous studies but is approximately 6% to 19% [2]. Symptoms of thyroid TB are non-specific. The patient may be asymptomatic or have symptoms of dysphonia, dysphagia, dyspnea, and rarely recurrent laryngeal nerve paralysis due to an expanding gland. Demonstrating acid-fast bacilli by staining confirms the diagnosis, but this stain is sometimes negative in tissue sections [4]. Furthermore, while thyroid function was thought to be normal in many cases of thyroid TB, a recent study has reported that patients with TB have a significantly higher risk of developing hypothyroidism than those without TB, although this study was not limited to thyroid TB [5]. Careful observation of thyroid hormones may also be necessary in patients with TB. In our case, a thyroid mass was not detected at the beginning of treatment, but it appeared during treatment. A fine needle aspiration cytology (FNAC) was performed, and the results showed a positive acid-fast bacteria smear but a negative culture. A slight decrease in TSH and recurrent laryngeal nerve paralysis were also observed, but as tuberculosis treatment was continued, the TSH level normalized, and the thyroid mass shrank. Considering the clinical course, it is considered to be thyroid TB as a paradoxical reaction. Paradoxical reaction in TB is common and is observed even in HIV-negative patients [6]. Some symptoms and clinical features, including paradoxical reactions, should be closely monitored during TB treatment.

The treatment of TB has developed, and a standard 6-month regimen is now recommended, comprising isoniazid, rifampicin, pyrazinamide, and ethambutol [7]. Rifampicin plays a crucial role as a key drug in this regimen. However, its clinical use is often complicated by notable adverse events. Additionally, rifampicin is recognized for its potential drug-drug interactions with various drugs. This is primarily due to rifampicin’s induction of multiple isoenzymes, particularly as a potent inducer of cytochrome P-450 (CYP) enzymes, including CYP1A2, CYP2B6, CYP2C19, CYP2C9, and CYP3A4, significantly impacting the metabolism of numerous drugs [8]. In comparison, rifabutin exhibits a narrow induction spectrum and 30% to 60% weaker CYP3A4 induction properties [9]. Consequently, rifabutin has been employed as a substitute for rifampicin in situations where rifampicin substantially reduces the plasma concentrations of co-administered drugs, such as HIV/tuberculosis co-infected patients taking protease inhibitors [10].

Besides potential drug-drug interactions, rifampicin is also associated with significant hematological adverse events such as neutropenia and thrombocytopenia, which are also observed with rifabutin [9,11]. Thrombocytopenia is generally defined as a platelet count less than 150 × 10^9^/L, although some propose a more clinically significant cut-off value of 100 × 10^9^/L [12]. A previous report has compiled cases of rifampicin-induced thrombocytopenia, while a cohort study on prophylactic rifampicin administration reported an incidence rate of 3.4% [11,13]. In contrast, large clinical trials indicate that severe thrombocytopenia occurred in 0.5% to 0.7% of patients at 300 mg/day of rifabutin [9]. Given the infrequency of such occurrences, directly comparing the superiority of these drugs in terms of drug-induced thrombocytopenia may be challenging. However, rifabutin can present itself as a compelling alternative to rifampicin, particularly for patients facing challenges with rifampicin use due to various reasons. Additionally, because of the rarity of miliary TB cases, there is limited evidence regarding the effectiveness of rifabutin in miliary TB treatment. Nevertheless, a previous report demonstrated the successful completion of treatment with rifabutin in a patient with extrapulmonary TB who experienced rifampicin-induced thrombocytopenia [14]. In our case, despite the persistent low platelet count even after discontinuation of rifampicin, rifabutin proved effective in treating the miliary TB patient who encountered rifampicin-induced thrombocytopenia. This case suggests the potential efficacy of rifabutin treatment in miliary TB patients facing challenges with rifampicin for various reasons.

## Conclusions

We report a case where thyroid tuberculosis manifested during miliary tuberculosis treatment. Close monitoring is essential, considering the possibility of thyroid TB occurrence. Furthermore, our case highlights the potential efficacy of rifabutin as a treatment for miliary TB, especially in patients facing rifampicin-induced thrombocytopenia during treatment.
